# Lipidome Investigation of Carnosine Effect on Nude Mice Skin to Prevent UV-A Damage

**DOI:** 10.3390/ijms241210009

**Published:** 2023-06-11

**Authors:** Beatrice Zoanni, Gilda Aiello, Anne Negre-Salvayre, Giancarlo Aldini, Marina Carini, Alfonsina D’Amato

**Affiliations:** 1 Department of Pharmaceutical Sciences, University of Milan, 20133 Milan, Italy; beatrice.zoanni@unimi.it (B.Z.); gilda.aiello@uniroma5.it (G.A.); giancarlo.aldini@unimi.it (G.A.); alfonsina.damato@unimi.it (A.D.); 2 Department of Human Science and Quality of Life Promotion, Telematic University San Raffaele, 00166 Rome, Italy; 3 Faculty of Medicine, Department of Biochemistry, INSERM U1297 and University of Toulouse, 31432 Toulouse, France; anne.negre-salvayre@inserm.fr

**Keywords:** skin, lipidome, UV-A, carnosine

## Abstract

The lipid profile of skin is fundamental in the maintenance of the protective barrier against the external environment. Signaling and constitutive lipids of this large organ are involved in inflammation, metabolism, aging, and wound healing, such as phospholipids, triglycerides, FFA, and sphingomyelin. Skin exposure to ultraviolet (UV) radiation results in a photoaging process that is an accelerated form of aging. UV-A radiation deeply penetrates the dermis and promotes damage to DNA, lipids, and proteins by increasing the generation of reactive oxygen species (ROS). Carnosine, an endogenous β-alanyl-L-histidine dipeptide, demonstrated antioxidant properties that prevent photoaging and modification of skin protein profiling, making carnosine a compelling ingredient to consider for use in dermatology. The aim of this research was to investigate the modification of skin lipidome after UV-A treatment in presence or not of topic administration of carnosine. Quantitative analyses based on high-resolution mass spectrometry of nude mice skin-extracted lipids resulted in several modifications of barrier composition after UV-A radiation, with or without carnosine treatment. In total, 328 out of 683 molecules showed significant alteration—262 after UV-A radiation and 126 after UV-A and carnosine treatment versus controls. Importantly, the increased oxidized TGs after UV-A radiation, responsible of dermis photoaging, were completely reverted by carnosine application to prevent the UV-A damage. Network analyses also showed that the production of ROS and the calcium and TNF signaling were modulated by UV-A and carnosine. In conclusion, lipidome analyses attested the carnosine activity to prevent the UV-A damage, reducing the lipid oxidation, the inflammation, and the dysregulation of lipid skin barrier.

## 1. Introduction

The skin is composed of multiple layers with distinct lipid content, each with unique and essential biological functions for maintaining skin properties and homeostasis [1]. The outermost layer, consisting of skin barrier lipids, is particularly susceptible to alterations. The skin barrier function is largely due to the stratum corneum (SC), the outer layer of the epidermis, particularly enriched in barrier lipids such as ceramides, with long acyl chains (C12-26), free fatty acids, cholesterol, and its esters, and cholesterol sulfate. These lipids fill the extracellular spaces and form an effective barrier to the environment, thus protecting the body against external factors such as UV radiation. However, the maintenance of a moist, pliable, and healthy skin barrier can be affected not only by the presence of these lipids. It also depends on their proper composition and organization within the SC. Indeed, a change in lipid composition, organization, and homeostasis has been associated with a number of skin diseases [2,3]. At the same time, membrane lipids found in the inner skin layers, such as glycerophospholipids, sphingomyelins, and cholesterol have been shown to impact the skin’s immune properties in both healthy and diseased states and play a crucial role in metabolism, aging, and response to UV radiation.

The solar ultraviolet radiation that reaches the Earth’s surface comprises about 5% of UVB (290–320 nm) and 95% of UV-A light (320–400 nm), and both are strongly associated with skin photoaging and tumorigenesis [4]. While the carcinogenicity of UV-B has been well established, UV-A has only been described as “potentially carcinogenic to humans”. Nevertheless, UV-A’s effects on the dermis have been widely explored, likely because UV-A is more efficient in reaching this skin layer than UVB which, on the contrary, affects only the outer layers. At the molecular and biochemical level, chronic exposure to UV-A progressively leads to a disorganization of the extracellular matrix (ECM), dysregulated autophagy, chronic inflammation, and reactive oxygen species (ROS) production, all of which contribute to skin aging and increase the risk of developing skin cancer [5,6]. These effects result in DNA, protein, and lipids damage. Among these, lipids play a critical role in the skin, including regulation and maintenance of the epidermal barrier, protection against external stimuli such as pathogens, allergens, and other xenobiotics, and preservation of the skin’s physical properties. However, these biological functions can be influenced by both the lipid composition of the various skin layers and the chemical changes that lipids may undergo after specific stimuli. For instance, it is known that cholesterol, phospholipids, free fatty acids, and squalene are targets for non-enzymatic lipid oxidation generated by UV through a free radical mechanism that leads to the formation of bioactive products. At the same time, UV has been shown to activate phospholipases, lipoxygenases, and cyclooxygenase which, in turn, generate a wide variety of fatty acid-derived mediators, with eicosanoid species being the most prominent [7]. However, despite being predominantly carcinogenic, UV-B radiation also exerts positive effects. Beyond the beneficial production of vitamin D, UV-B presents immunomodulatory effects, both locally and systemically, which have been less explored but can potentially be applied for the therapeutic treatment of autoimmune and inflammatory disorders. In addition, UV radiation is able to regulate overall skin homeostasis by the activation of local neuroendocrine axes, thus regulating various physiological processes and responses to environmental stresses [8].

In this scenario, numerous natural compounds such as strawberry-based formulations and natural antioxidants such as rutin were detected as protecting agents against UV-induced skin damage [9]. Among them, carnosine, an endogenous β-alanyl-L-histidine dipeptide, demonstrated interesting antioxidant and carbonyl scavenger properties that significantly prevent photoaging, making carnosine a compelling ingredient to consider for use in dermatology [10]. At the molecular level, carnosine was found to exert inhibitory effects on the modification of elastin as well as the process of fibroblast senescence induced by 4-Hydroxynonenal (HNE) or acrolein [11]. Carnosine readily reacts with aldehydes such as acrolein or HNE to form nonreactive adducts, preventing the process of protein modification and subsequent alteration of cells and tissues [12,13]. More recently, carnosine has shown promising results in the prevention of oxidative stress modulation on the scaffold-free human dermis spheroids irradiated with and without UV-A [14]. Additionally, proteomic studies based on the UV-A-irradiated nude mice skin and fibroblast spheroids demonstrated that carnosine was effective in preventing the changes of proteome [5]. 

Although there are many in vivo and in vitro studies concerning the effect of carnosine on the proteome, the studies on the modulation of lipid composition by UV-A exposure are still limited. In this context, lipidomics occupies a predominant position in dermatologic research and represents a suitable technique for answering these biological questions. Lipidomics stands for the large-scale profiling and characterization of lipid species in a biological system using high resolution mass spectrometry (coupled or not with liquid chromatography) and provides a sensitive and specific tool to identify and quantify skin lipids. Lipidomic profiling of biological samples is nowadays achieved by either targeted or untargeted approaches. Untargeted lipidomics provides a more holistic view of the lipidome, although the data processing can be quite elaborate. In this work, we employ an untargeted lipidomics approach to determine significant changes in skin lipidome after UV-A exposure with and without carnosine treatment. 

## 2. Results and Discussion

We previously reported that hairless mice exposed to UV-A (20 J/cm^2^, up to 600 J/cm^2^) daily developed clinical patterns of photoaging and lipid peroxidation that were partly protected when the animals were previously treated by carnosine [11]. In the present study, we aimed to investigate the potential role of carnosine in mitigating the effects of UV-A exposure on skin lipidome in hairless mice. For this purpose, lipidomic studies were carried out on whole skin homogenates recovered from skin samples of UV-A-exposed hairless mice. Untargeted lipidomic analyses supported the detection of 2405 features of which 937 with annotated reference spectra matched against the Lipidblast database either in positive or negative ionization mode. The subsequent manual identification allowed the identification of 683 lipid molecular species (Table 1). Among the identified lipid species, triacylglycerols (TGs) demonstrated the higher abundance covering 21.8% of the identified lipidome, followed by ceramides (Cer, 15.2%), sphingomyelin (SMs, 5.3%), phosphatidylcholines (PCs, 5.3%), and fatty acids (FAs, 5.0%). Diacylglycerols (DGs, 6.4%), cholesterol esters (CEs, 2.2%), phosphatidylethanolamine (PEs, 1.8%), lyso-phosphatidylethanolamine (LPEs), lyso-phosphatidylcholines (LPC), and phosphatidylinositol (PIs) complete the qualitative description of the most abundant lipid classes identified in the analysis.

**Figure 1:**
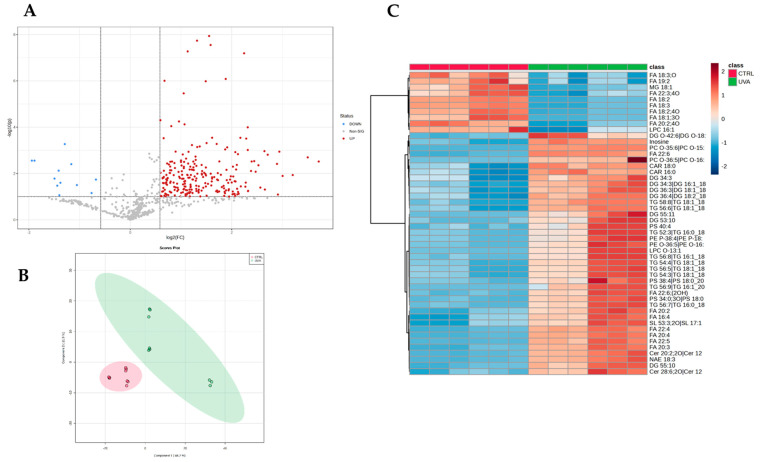
(**A**) Volcano Plot of UV-A vs. CTRL. In red are the features significantly altered (fold change (RATIO) > 1.5, adjusted *p*-value < 0.05; (**B**) Scoring plots reconstructed using PLS-DA. The red group corresponds to the control samples; the green group corresponds to those exposed to UV-A; (**C**) Hierarchical clustering heat map of the 40 most significantly different lipids between the two groups. Refer to Table Supplementary for full lipid names. The color-coded map illustrates abundance values, where red color indicates relatively high expression levels, while blue indicates relatively low expression levels. The sample data are arranged in rows, and the features investigated are presented in columns.

Once the identified compounds were normalized, the lipidome profiles of each experimental condition were compared. Considering all groups, 328 out of 683 features resulted significantly altered at least between two conditions based on the one-way ANOVA test (adjusted *p*-value < 0.05, post-hoc analyses using Fisher’s LSD). The volcano plot analysis, resulted from two-sided *t*-test (FC > 1.5, *p*-value < 0.05), showed that the UV-A exposure induced a significant modulation of 262 features (Figure 1A) vs. controls belonging to cholesterol esters (CE; *n* = 7), acylcarnitines (CAR; *n*= 6), ceramides (Cer; *n* = 48), sphingomyelins (SM; *n* = 23), triacylglycerols (TG; *n* = 73), and diacylglycerols (DG; *n* = 20). Similarly, phospholipids classes, i.e., phosphatidylcholines (PC), lyso-phosphatidylcholines (LPC), phosphatidylethanolamines (PE), lyso-phosphatidylethanolamines (LPE), phosphatidylinositols (PI), phosphatidylglycerols (PG), and phosphatidylserines (PS), were also found to be affected by UV-A exposure. Partial least squares-discriminant analysis (PLS-DA) (Figure 1B) and unsupervised clustering demonstrates the pattern where the mice irradiated with UV-A were clearly separated from control ones (Figure 1C).

TGs and DGs content remodeling have important biological effects. The build-up of TGs in non-adipose tissue is linked to lipid toxicity while DGs function as second messengers and are implicated in the inflammatory response [15]. However, we also observed an increase in oxidized TGs levels (oxTGs) (TG 50:4;O2 log_2_ratio = 1.11, TG 52:4;O2 log_2_ratio = 1.65) that together may contribute to altering skin homeostasis. In addition, several ether-linked glycerolipids were detected and found to be upregulated. Of note, the increase in cholesterol sulfate (log_2_ratio = 0.51) and cholesterol ester levels (CE 32:1 log_2_ratio = 1.91, CE 36:1; log_2_ratio = 1.26) represents another evidence of the marked dysregulation of the skin physiological processes. Significant changes in phospholipid profiles were also observed after UV-A exposure. These changes include the upregulation of PC species such as PC 34:0 (PC 16:0_18:0; log_2_ratio = 0.97), PC 36:4 (PC 16:0_20:4; log_2_ratio = 1.33), and LPC (LPC 20:4; log_2_ratio = 0.78, LPC 22:0; log_2_ratio = 0.57). LPC is synthesized by the hydrolysis of PC through the action of phospholipase A2 (LPA2). While LPC is a biologically active lipid, it is produced only under certain pathological conditions. Indeed, the concentration of LPC is tightly regulated to ensure optimal cellular function because excessive amounts of LPC can cause cell lysis and death. Furthermore, we found interesting changes in the relative abundance of PS species such as PS 36:2 (PS 18:1_18:1; log_2_ratio = 0.60) and PS 38:4 (PS 18:0_20:4; log_2_ratio = 0.72). Increasing evidence shows that these phospholipids play a role in several metabolic pathways in the body, including those involving the metabolism of arachidonic acids, linoleic acids, and α-linoleic acids [16].

Interestingly, the free fatty acids profile also appears to be significantly modulated by UV-A exposure. FFAs are known to influence cell proliferation, both in vivo and in vitro. Upon exposure to UV-A, we observe that the relative abundance of oleic acid FA18:1 (log_2_ratio = −1.04), linoleic acid 18:2 (log_2_ratio = −1.52), and linolenic acid 18:3 (log_2_ratio = −1.47) decreases while that of some PUFAs such as FA20:2 (log_2_ratio = 0.65), FA20:3 (log_2_ratio = 1.04), FA20:4 (log_2_ratio = 1.68), FA22:4 (log_2_ratio = 1.42), FA22:5 (log_2_ratio = 1.52), and FA22:6 (log_2_ratio = 1.61) increases. Several studies have indicated how PUFAs may play a key role in skin physiology and pathology by acting as real bioactive lipids. Linoleic acid is the most predominant polyunsaturated fatty acid (PUFA) found in the epidermis, accounting for approximately 12% of fatty acids. Impaired epidermal barrier function is associated with LA deficiency. For example, ceramide and sphingolipid species rich in LA are reported to be critical for preserving skin hydration, preventing trans-epithelial water loss, and maintaining the epidermal barrier. In contrast, the upregulation of arachidonic acid and other related PUFA such as docosahexaenoic acid (22:6, DHA), as precursors of eicosanoids, may be indicative of the induction of a UV-inflammatory response [17]. At the same time, due to the high number of double bonds, PUFAs may be more susceptible to lipid peroxidation and may increase the risk of oxidative stress. Furthermore, the upregulation of acylcarnitines such as CAR 16:0 (log_2_ratio = 0.68), CAR 16:1 (log_2_ratio = 0.58), CAR 18:0 (log_2_ratio = 0.58), and CAR 18:1 (log_2_ratio = 0.70) confirmed UV-A effects in altering fatty acids metabolism.

Among the other lipid classes altered, sphingolipids and ceramides were found to be upregulated. The findings suggest that ceramides were more active in the mice exposed to UV-A compared to those in the control group. Among them, concentrations of Cer(d42:1) (log_2_ratio = 0.76), Cer(40:0) (log_2_ratio = 1.02), Cer(34:1) (log_2_ratio = 0.69), and ultra-long chain Cers were significantly increased. The altered ceramide expression profiles can lead to decreased extracellular lipid matrix density and organization driving progressive fibrosis [18,19], as well as to a persistent generation of inflammatory cytokines and chemokines, such as IL-1α and TNF-α, which exacerbate the inflammatory response [20].

### 2.1. The Protecting Role of Carnosine on Skin Dermis Lipidome

**Figure 2:**
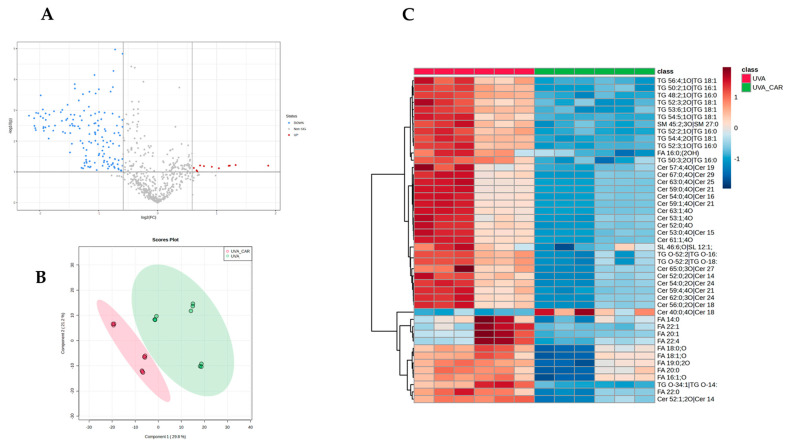
(**A**) Volcano Plot of UV-A_CAR vs. UV-A; (**B**) Scoring plots reconstructed using PLS-DA; (**C**) Hierarchical clustering heat map of the 40 most significantly different lipids between the two groups. Refer to Table Supplementary for full lipid names.

To investigate the potential role of carnosine in mitigating the effects of UV-A exposure on skin lipidome, we conducted a comparison analysis between the UV-A plus carnosine group (UV-A_CAR) and the UV-A group without carnosine (UV-A). The treatment with carnosine induced a significant modulation of 126 features (Figure 2A) with 60 of them showing an opposite trend compared with the previous comparison (UV-A vs. control). These findings suggest that the treatment holds the capacity to reverse the dysregulation of the lipidomic profile, thus improving the skin’s physiological processes required for skin barrier integrity, stability, and function. Partial least squares-discriminant analysis (PLS-DA) (Figure 2B) and clustering (Figure 2C) confirmed the differences in the lipidomic profile between the two groups. Among those most differentially regulated by the treatment, we found lipids belonging to cholesterol esters (CEs; *n* = 5), triglycerides (TGs *n* = 17), fatty acids (FAs; *n*= 4), and ceramides (Cer; *n*= 28).

We found that carnosine supplementation preserved cholesterol metabolism as suggested by the reduction in cholesterol (log_2_ratio = −1.05) and long fatty acyl chains CEs levels (CE 32:1, CE 34:1, CE 36:1) (log_2_ratio = −0.95, log_2_ratio = −0.91, log_2_ratio = −0.76) that were previously found to be upregulated, thus improving membrane plasticity and skin metabolic remodeling. Further than CEs, carnosine also decreases the levels of other neutral membrane lipids such as TGs enhancing the skin barrier function and reducing the lipid toxicity associated with their overload. Interestingly, oxidized TGs levels (TG 16:0_16:1_18:3;1O; log_2_ratio = −0.86 and TG 18:1_18:1_18:2;1O; log_2_ratio = −1.30) seem to be the most affected by the treatment, confirming the carnosine antioxidant effect and its ability to modulate the unbalanced redox state that the skin undergoes after prolonged UV-A exposure. As further evidence of this effect, carnosine treatment also leads to a significant reduction in the levels of arachidonic acid (log_2_ratio = −0.5) and other upregulated PUFAs while it does not appear to have an effect on the levels of linoleic acid, which remain unaffected. Moreover, carnosine decreased several ceramides including Cer 18:0;O2/22:0 (log_2_ratio = −0.56) and ultra-long chain ceramides. Interestingly, the treatment seems to affect principally the levels of α-hydroxy-N-stearoyl phytosphingosine ceramides (Cer AP). This aids in improving inflammatory response and proper skin hydration and, on the other hand, results in better skin plasticity, enhanced barrier function, and protection against external injuries.

The identification and quantification of lipid species differentially regulated for each condition (control, UV-A_carnosine, UV-A) were followed by an analysis of the lipid network to describe the functional modules and pathways altered by UV-A radiations with/without carnosine.

### 2.2. UV-A Exposure Evokes the Activation of Ceramide and Sphingosine-1-Phosphate Signaling

**Figure 3:**
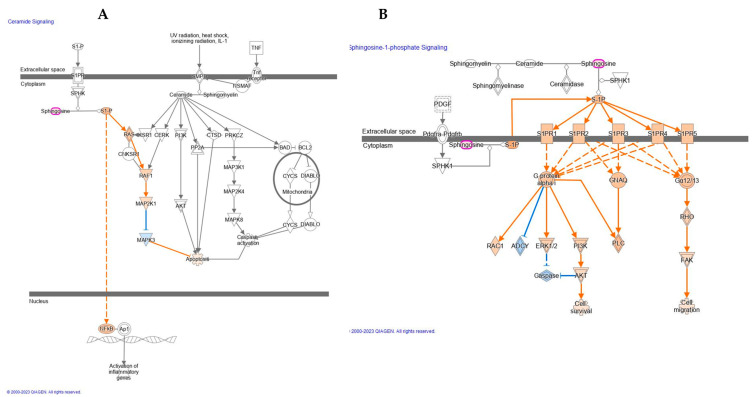
Activation of Ceramide (**A**) and Sphingosine-1-phosphate signaling (**B**) after UV-A exposure. Orange represent activated genes whereas the blue one the deactivated.

Network analyses showed several changes in skin dermis of nude mice lipidome evoked by UV-A exposure and carnosine treatment. UV-A damaged the skin by the alteration of specific functional modules, such as the generation of reactive oxygen species, cell proliferation of tumor cell lines, quantity of Ca^2+^, and concentration of lipid (Table 2) as well as the activation of ceramide and Sphingosine-1-phosphate signaling (Figure 3).

Upon the UV-A treatment, our results demonstrate the activation of the ceramide signaling pathway. Specifically, sphingosine was found upregulated which results in the activation of sphingosine-1-phosphate (S1P). A wide range of studies demonstrated that S1P is involved in the pathogenesis of diverse inflammatory skin diseases, specifically in atopic dermatitis, type I and type IV allergic skin inflammation, psoriasis, and scleroderma [21,22]. In addition, it may play an important role in the progression of melanoma and in the regulation of stress-induced apoptosis [23]. Here, as reported in Figure 3A, the activation of S1P indirectly induces the upregulation of NFkB. The majority of recent studies, however, have implicated an important role of G protein-coupled S1P receptors in mediating the biological functions of S1P. These receptors integrate signals with specific signal transduction pathways, e.g., activation of the extracellular signal-regulated kinase (ERK1/2) as reported in Figure 3B. Receptor activation also leads to other intracellular changes, such as decreased levels of cyclic AMP (cAMP) and increased production of inositol phosphate and level of Ca^2+^ as observed in our study and mentioned above. 

### 2.3. The Effect of Carnosine on ROS Generation and Calcium Signaling Pathway Modulation Evoked by UV-A

**Figure 4:**
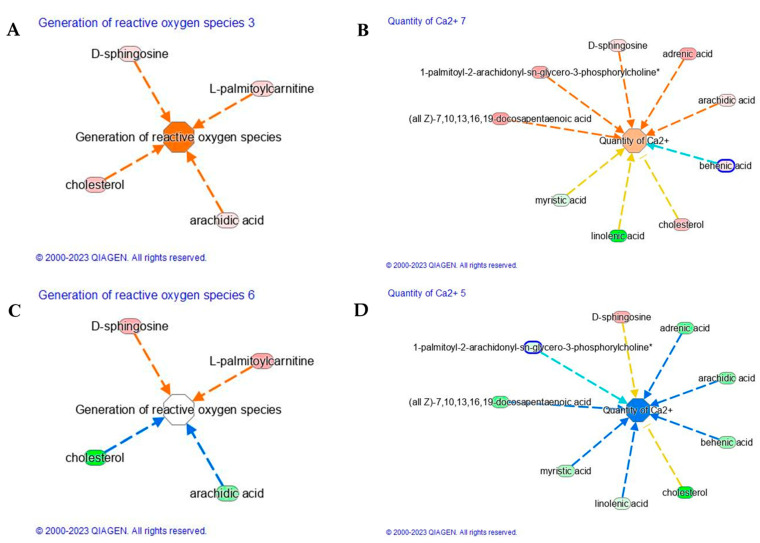
Generation of reactive oxygen species pathways in (**A**) UV-A vs. Control and (**C**) UV-A_CAR vs. UV-A. Quantity of Ca^2+^ signaling in (**B**) UV-A vs. Control and (**D**) UV-A_CAR vs. UV-A. The increased genes are in red, and those decreased are in green. The color intensity is positively related to the up- or down-gene’s regulation; the orange line leads to activation, blue lines for deactivation, yellow lines for findings inconsistent with state of downstream molecule, grey line for effect not predicted. (For interpretation of the references to color in this figure legend, the reader is referred to the Web version of this article). Asterisk indicates that multiple identifiers in the dataset file map.

UV-A-induced ROS initiate diverse responses in keratinocytes including the synthesis of proinflammatory arachidonic acid metabolites, which are believed to contribute to promotion in the development of UV-induced nonmelanoma cancers. Our study demonstrates that UV-A induced ROS generation (Z-score 1.9, Figure 4A), due to the upregulation of cholesterol, arachidic acid, D-sphingosine, and L-palmitoylcarnitine (Table 2). The saturated long-chain fatty acids (SLCFAs) such as arachidic (C20:0), as well as myristic (C14:0), palmitic (C16:0), stearic (C18:0), and behenic acid (C22:0), are generally regarded to promote the proliferation and differentiation of inflammatory T cell, astrocyte, and microglia/macrophage subsets [24,25]. FAs are not merely substrates for energy production but appear as important modulators of various cellular functions including inflammation. Saturated FAs, but not unsaturated FAs, are believed to directly increase intracellular NF-kB signaling via activation of Toll-like receptors (TLRs). Particularly, the TLR4 activation by SLCFAs increases the expression of a number of inflammatory genes in adipocytes by a nuclear factor κB–dependent mechanism [26]. Several studies confirm the induction of ROS upon UV-A treatment, whereas fewer report the protective effect of carnosine applications. Carnosine treatment was able to reduce the level of ROS whose functional module was characterized by a lower Z-score. As reported in Figure 4C, the main lipids involved in the generation of reactive oxygen species, such as cholesterol and arachidic acid, were found to downregulate upon carnosine treatment. The reduction of arachidic (C20:0) acid upon carnosine application ameliorates the generation of ROS. 

Furthermore, UV-A evokes Ca^2+^ signaling pathway characterized by lower Z-score (−1.65) than that observed after UV-A exposure (Z-score = 0.65, Figure 4B). Calcium ions (Ca^2+^) and their concentration gradient in the epidermis are essential in regulating many skin functions, including keratinocyte differentiation, skin barrier formation, and permeability barrier homeostasis. The main lipids, here observed, to be involved in the activation of Ca^2+^ signaling were cholesterol, arachidic acid, adrenic acid, D-sphingosine, 1-palmitoyl-2-arachidonyl-sn-glycero-3-phosphorylcholine, and docosapentaenoic acid (DPa). Short-term exposure to UV-A radiation can directly injure our skin through inflammatory response and indirectly through oxidative stress, triggering polyunsaturated fatty acid (PUFA) peroxidation in the skin-cell membrane. The direct involvement of DPA was recently assessed on human keratinocytes (HaCaT) which were exposed to low dose (5 J/cm^2^) and high dose (20 J/cm^2^) of UV-A [27]. A dose of 20 J/cm^2^ UV-A stimulated a significant amount of arachidonic acid and docosahexaenoic acid (DHA) after 24 h exposure, indicating that UV-A radiations seem to accumulate PUFA and disrupt normal PUFA metabolism even after 24 h. The accumulation of PUFA may alter the function of skin cells, for example, by increasing the fluidity of cell membrane and lipid peroxidation in skin [28,29,30]. Moreover, it has been reported that UV-A activates a G-protein coupled signaling pathway that leads to intracellular calcium (Ca^2+^) mobilization, a condition involved in migration, invasion, and metastasis of different types of cancers [31]. Ca^2+^ influx, activated by UV-A exposure, can lead to cellular damage by contributing to the development of skin aging and cancer. However, carnosine treatment induced a reverse effect on Ca^2+^ signaling pathway by modulating negatively the adrenic (C22:4), arachidic (C20:0), behenic, or docosanoic (C22:0) and cholesterol (Figure 4D). Carnosine seems to restore the level of linoleic acid, significantly reduced by UV-A. Linoleic acid has been suggested to play a role in calcium signaling in skin. It is an essential fatty acid that is a precursor for the synthesis of ceramides, which have been shown to regulate calcium signaling in keratinocytes. In addition, several studies have suggested that linoleic acid may have anti-inflammatory effects on skin. Therefore, the presence or absence of linoleic acid in the skin could impact calcium signaling and overall skin health. Similarly, UV-A negatively affects the level of miristic acid, compromising the balance of saturated and unsaturated fatty acids, which plays an important role for maintaining the barrier function of the skin and preventing moisture loss after UV-A exposure. 

### 2.4. Effect of Carnosine on TNF Dysregulation Evoked by UV-A Exposure

**Figure 5:**
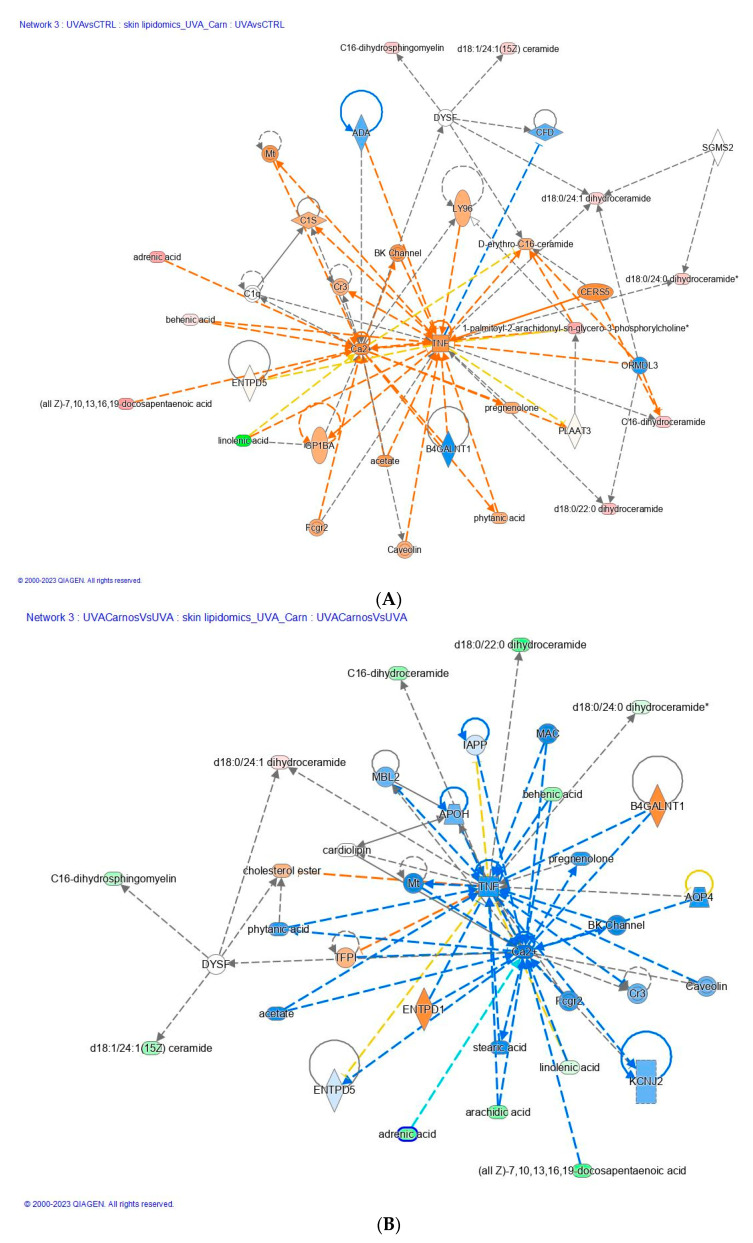
TNF and Ca^2+^ dysregulation after (**A**) UV-A exposure and (**B**) after carnosine treatment. The increased genes are in red, and those decreased are in green. The color intensity is positively related to the up- or down-gene’s regulation; the orange line leads to activation, blue lines for deactivation, yellow lines for findings inconsistent with state of downstream molecule, grey line for effect not predicted. Asterisk indicates that multiple identifiers in the dataset file map.

UV-A radiation can stimulate the production of tumor necrosis factor (TNF) in the skin as reported in Figure 5A. Its production is known to increase in response to various types of stress, including exposure to UV-A radiation, and induces the development of various skin conditions, including photoaging, skin cancer, and inflammatory skin diseases such as psoriasis.

TNF activates various signaling pathways in the skin, leading to the production of other pro-inflammatory cytokines and the activation of immune cells, which can contribute to tissue damage and disease progression. Therefore, our data confirm that UV-A induced TNF production, which is considered to be an important factor in the harmful effects of UV-A radiation on the skin. The activation of the TNF pathway is completely reversed by the protective anti-inflammatory action of carnosine as shown in Figure 5B. Carnosine has been shown to have anti-inflammatory and antioxidant effects since it was able to reduce the production of TNF in the skin. Our data confirm the effect observed to reduce the expression of TNF in animal models of inflammatory skin diseases such as atopic dermatitis. The mechanism by which carnosine exerts its anti-TNF effects is not entirely clear, but it is thought to involve the inhibition of various signaling pathways that are involved in TNF production.

A variety of lipids are involved in the activation of TNF signaling as shown in Figure 5, and targeting these lipids or their downstream signaling pathways may have therapeutic potential for the treatment of TNF-mediated skin conditions. A key role is played by d18:0/22:0 dihydroceramide, which is implicated in the regulation of TNF signaling in the skin. Dihydroceramides are precursors to ceramides, which are important components of the skin lipid barrier and are involved in various signaling pathways, including TNF signaling. Our findings suggest that UV-A induces the upregulation of d18:0/22:0 dihydroceramide, which promotes the formation of a pro-inflammatory complex called the ceramide synthase complex. This complex is involved in the production of ceramide, a lipid that plays a key role in various cellular processes, including apoptosis and inflammation. Therefore, d18:0/22:0 dihydroceramide may contribute to the regulation of TNF-mediated inflammatory responses. The exposure to UV-A radiation can lead to an increase in d18:0/22:0 dihydroceramide levels in response to pro-inflammatory signaling pathways. The UV-A generation of reactive oxygen species (ROS) activates sphingomyelinases, leading to the breakdown of sphingomyelin and to the production of ceramides, such as d18:0/22:0 dihydroceramide [32]. This increase in ceramides can then activate TNF signaling, leading to the expression of pro-inflammatory cytokines and chemokines and contributing to skin inflammation and damage.

On the other side, carnosine has been shown to play a protective role against the harmful effects of d18:0/22:0 dihydroceramide in the skin. Several forms of DhCer (C22:0 and C24:0) (Figure 5, Table S1) induced cytotoxicity through a caspase-independent mechanism, mixed cell death with increased autophagy [33]. The role of DhCer as inducers of autophagy has been further confirmed by others in several cellular models [34,35].

Strongly connected to inflammation was the c16 dihydrosphingomyelin (dhSM), found upregulated after UV-A exposure. Specifically, UV-A radiation increased the levels of c16 dhSM in the skin, which may contribute to the disruption of the skin barrier and to an increase in the expression of pro-inflammatory genes.

Carnosine, on the other hand, has been found to show antioxidant and anti-inflammatory properties, which can help to counteract the negative effects of d18:0/22:0 dihydroceramide and dhSM. Studies have shown that the topical application of carnosine can help to reduce skin inflammation, improve skin hydration, and prevent damage to the skin [36]. Further research is needed to fully understand the effects of carnosine on dhSM in skin. Overall, carnosine may be a useful ingredient in skincare products aimed at improving skin health and preventing skin disorders.

## 3. Materials and Methods

### 3.1. Experimental Treatment

Lipidomic studies were carried out on skin samples recovered from our previous study [11]. The experimental protocol No. 12/1048/10/13 was conducted in accordance with French legislation and approved by the medical ethical committee for all described studies. The experiment involved the use of Albino hairless mice Skh:hr-1 (6 weeks old) obtained from Charles River Laboratories in Saint Germain sur l’Arbresle, France. Three animals were assigned to each condition, with one control non-irradiated group, one group daily exposed (on the whole back) to UV-A radiation for six weeks (2 h exposure per day, 5 days a week, the single dose of UV which was 20 J/cm^−2^ per day resulting in a total of 600 J/cm^2^ at the end of the experiment), one group daily exposed to UV-A and topically treated with carnosine (1% in polyethylene glycol PG) after exposure, and one group exposed to UV-A and treated with PG alone. This protocol did not induce any visible signs of photo-irritation and phototoxicity [11]. After sacrificing the animals, skin samples from the backs of the mice were obtained and stored at −80 °C until their use. More precise experimental conditions and clinical features of skin photoaging and protection by carnosine are detailed by Larroque-Cardoso et al. [11].

### 3.2. Sample Preparation

The study was conducted on whole skin samples without separation of the different skin layers. First, 80 mg of each skin sample was homogenized by glass bead beating (3 cycles, 60′′, 350 rpm) in 300 μL of i-PrOH/H2O (50/50 *v*/*v*), and 20 μL was taken for BCA protein assay. An aliquot corresponding to 100 μg of proteins was quickly spiked with 10 μL of SPLASH^®^ LIPIDO-MIX^®^ and left into ice for 15 min. Another aliquot of each sample was transferred to a separate vial to prepare a pooled QC sample. The lipids extraction was performed by the standard MTBE protocol with MTBE/methanol/water (10:3:2.5, *v*/*v*/*v*) as extraction solvents ratio [37]. All solvents contained BHT 0.1% *w*/*v* to prevent unwanted oxidation. Briefly, 700 μL of MTBE/MeOH (10:3) was added to each sample, vortexed for 5 s, and incubated for 1 h at 4 °C in Thermomixer. The phase separation was induced by adding 140 μL of H2O, vortexing for 5 s, and 15 min of incubation (4 °C, 210 rpm). Once centrifugated (4 °C, 15 min, 13,400× *g*), the upper phase was collected into a new tube. The upper phase was then dried under vacuum (Eppendorf concentrator 5301, 1 mbar). Before the LC-MS analyses, lipids extracts were dissolved in 200 μL i-PrOH/ACN (90/10, *v*/*v*, with ammonium acetate 10 mM and 0.1% formic acid) and vortexed. 

### 3.3. High-Resolution Mass Spectrometry Analysis (nLC-HRMS) and Data Analysis

All samples have been analyzed at UNITECH OMICs (University of Milano, Italy) using ExionLC™ AD system (SCIEX) connected to ZenoTOF™ 7600 System (SCIEX) equipped with Twin Spray Turbo V™ Ion Source with ESI Probe. Chromatographic separation was achieved on a Kinetex^®^ EVO C18 (Phenomenex) 100 (Length) × 2.1 mm (ID) × 1.7 µm (Particle Size) using mobile phase A (H_2_O/ACN (60/40, *v*/*v*, with ammonium acetate 10 mM and 0.1% formic acid) and mobile phase B (i-PrOH/ACN (90/10, *v*/*v*, with ammonium acetate 10 mM and 0.1% formic acid) at a flow rate of 400 µL/min. The column and autosampler temperatures were set at 45 °C and 15 °C, respectively. The sample injection volume was 5 µL. The following gradient profile was used: 0.00 min, 45% B; 2.00 min, 45% B; 12.00 min, 97% B; 17.00 min, 97% B; 17.10 min, 45% B; 20.00 min, 45% B. MS spectra were collected over an m/z range of 140–1500 Da, operating in IDA^®^ mode (Information Dependent Acquisition). The collision energy was set at 35 (CES 15). The polarity was ESI positive/negative with consecutive injections. Three technical replicates (LC-MS/MS runs) were performed. QC samples were run at the beginning of the sequence, during the sequence, and at the end of the sequence. MS-Dial software (RIKEN, version 4.90) [38] was used to process the MS data. This involved peaks detection, MS2 data deconvolution, lipid identification, and alignment of peaks through all the samples. For identification, a cut-off value of 80% was selected. Relative quantification was based on the determination of the peak intensities for each lipid correctly identified and then normalized by the intensity of the used ISTD to the corresponding lipid class. Normalized peak intensities were then exported to Excel for statistical analysis by means of Metaboanalyst version 5.0 [39]. Finally, the lipid molecules showing significant changes were identified with unique IDs corresponding to the human metabolome database (HMDB) and subjected to network analysis using Ingenuity Pathways Analysis software (IPA, Qiagen, www.qiagen.com, USA).

## 4. Conclusions

In conclusion, our untargeted lipidomic study provides a detailed and precise characterization of the changes in the lipidomic profile of murine skin upon prolonged UV-A exposure and treatment with carnosine. Our findings indicate that UV-A radiation leads to profound alterations in skin barrier composition primarily affecting the levels of oxidized and neutral triglycerides, ceramides, and fatty acids, which together play a central role in maintaining skin barrier function. These changes lead to several detrimental effects on the skin, including dermal photoaging, chronic inflammation, and increased production of reactive oxygen species (ROS) as well as tumor necrosis factor (TNF). To prevent UV-A damage, skin treatment with carnosine was shown to be effective in regulating ROS and TNF generation induced by UV-A, confirming its antioxidant and anti-inflammatory properties and enhancing the skin physiological processes essential for its barrier integrity and stability. Beyond these observations, the high sensitivity of our lipidomic approach lays the foundations for future research aimed at gaining a more comprehensive understanding of the skin lipidome changes upon disease and treatment as well as evaluating the protective potential effect of agents that enhance skin health and prevent skin disorders. Further investigation will be dedicated to a more detailed study of oxidized lipids.

## Figures and Tables

**Table 1:** Summary of identified lipids.

A					
	Number of Lipids	%		Number of Lipids	%
Identified Features in MS-DIAL	685		Identified Features in MS-DIAL		
TG	149	21.8	PE	12	1.8
Cer	104	15.2	EtherPC	11	1.6
DG	44	6.4	LPC	11	1.6
PC	36	5.3	PI	11	1.6
SM	36	5.3	PS	11	1.6
FA	34	5.0	EtherPE	10	1.5
OxFA	30	4.4	MG	10	1.5
OxTG	29	4.2	NAE	8	1.2
EtherTG	25	3.6	CL	7	1.0
CE	15	2.2	CAR	6	0.9
HexCer	14	2.0	SHexCer	6	0.9
SL	13	1.9	LPE	5	0.7

**Table 2:** Functional modules evoked by UV-A exposure and protective treatment with carnosine. Positive z-score indicates the activation pathway; negative z-score indicates the downregulation of the pathway (the analysis was performed by IPA (Qiagen, Venlo, The Netherlands) using the quantitative LFQ dataset derived from statistical analysis of MS/MS data).

Categories	Functions Annotation				
		UV-A vs. Ctrl		UV-A_CAR vs. UV-A	
		*p*-Value	Z-Score	*p*-Value	Z-Score
Free Radical Scavenging	Generation of reactive oxygen species	3.02 × 10^−2^	1.98	3.02 × 10^−2^	0.00
Cellular Development, Cellular Growth and Proliferation	Cell proliferation of tumor cell lines	4.82 × 10^−2^	0.99	4.82 × 10^−2^	−0.261
Cell Signaling, Molecular Transport, Vitamin and Mineral Metabolism	Quantity of Ca^2+^	1.49 × 10^−5^	0.65	1.49× 10^−5^	−1.622
Lipid Metabolism, Molecular Transport, Small Molecule Biochemistry	Concentration of lipid	1.20 × 10^−2^	0.21	-	-
Lipid Metabolism, Molecular Transport, Small Molecule Biochemistry	Concentration of fatty acid	1.21 × 10^−2^	−1.704	1.21 × 10^−2^	−0.755

## Data Availability

We will share raw data upon request.

## Supplementary Materials

The following supporting information can be downloaded at: https://www.mdpi.com/article/10.3390/ijms241210009/s1.
